# Rosalind Farnam Dudden (1944-2023)

**DOI:** 10.5195/jmla.2024.1922

**Published:** 2024-07-01

**Authors:** Margaret Moylan Bandy

**Affiliations:** 1 mbandy81@gmail.com New Harmony, IN

**Figure:**
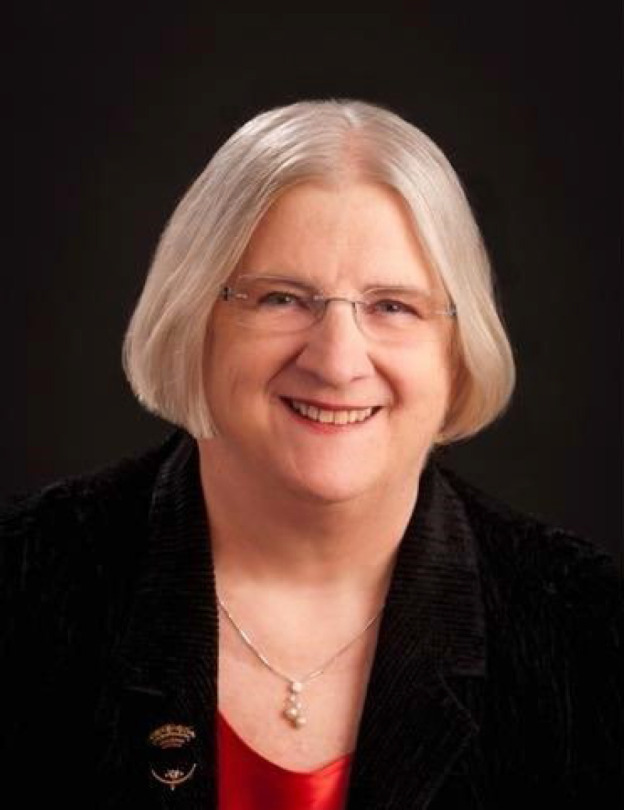


Rosalind “Roz” Farnam Dudden, AHIP, FMLA, died September 27, 2023 [1] at Lutheran Hospice in Wheat Ridge, Colorado. Her daughter, Laura Dudden, was with her, as was the love of her family and many friends.

To summarize any life in just a few words is impossible. Fortunately, Roz's oral history [2] gives a full picture of her professional life, in her own words. Roz's reflection before the interview questions is also worth reading as it provides key insights on her approach to her life and work.

A lengthy list of Roz's accomplishments and many awards, including receipt in 2013 of the Medical Library Association's (MLA's) highest honor, the Marcia C. Noyes Award, does not completely convey how Roz was regarded by her colleagues in MLA and other professional organizations. Betsy Humphreys, former deputy director of the National Library of Medicine, noted “Roz was indeed an outstanding colleague, great innovator, and major contributor to our profession. Her positive attitude and good humor made her a pleasure to work with. She was always part of the solution—never part of the problem” [3].

Early MLA activity included her affiliation with the Hospital Libraries Section (HLS), becoming its treasurer in 1977. Her leadership as chair of the Hospital Library Standards and Practices Committee helped produce the first edition of the MLA Hospital Library Standards, published in 1984. In that same year, the section presented her with an award for her efforts which they called Resolution of a Debt of Gratitude. Roz chaired HLS for the 1987/1988 association year, developed and maintained the HLS website, and chaired the HLS Web Site Task Force from 1995 to 1998. While providing technology leadership to HLS, she also chaired the Consumer and Patient Health Information Section (CAPHIS) Web Site Task Force from 1995 to 1998.

As an early adopter and promoter of technology for hospital libraries, innovation and collaboration with colleagues were hallmarks of Roz's activities. In 1980, when she first heard of the concept of an automated integrated library system, she organized a committee to explore how a group of Denver hospital libraries might share such a system. In 1983, the group issued a request for a quote from several vendors and the University of Colorado Health Sciences Center (UCHSC) Denison Memorial Library. The UCHSC proposal was accepted, and even though Roz was the driving force for this project, her institution would not fund her library's participation in the first group that joined the system.

Undaunted by the previous setback, and now serving as a librarian at a different organization, Roz established a new group of libraries to seek funding through an NIH/NLM Information Systems Grant in 1993. Members of the first group joined the funding effort to expand their existing programs, and $219,014 was awarded to the seven-member collaborative. In 1995, Roz received the Frank Bradway Rogers Information Advancement Award in recognition of her leadership and technological achievements.

Roz directed two Denver hospital libraries: Mercy Medical Center (1971-1986) and National Jewish Health (1986 to retirement in 2011). Her contributions to colleagues in the Colorado Council of Medical Librarians (CCML) included resource sharing activities. She helped create the first union list of serials in 1977 and remained involved as either chair or a committee member for fifteen subsequent editions. Roz inspired hospital librarians to get involved with Internet activities. She created Denver's first hospital website in 1995, taught HTML classes, and presented talks explaining the web's potential to many non-library groups.

In 1998, Roz was elected to the MLA Board of Directors. As a Board member and liaison to MLANET, she encouraged the development of technology to meet the needs of MLA members. During this time, Roz also worked tirelessly on the MLA Benchmarking Network initiative. In 2003, she received the MLA President's Award and attained MLA Fellowship status.

Among more than thirty publications, Roz authored the bestselling Using Benchmarking, Needs Assessment, Quality Improvement, and Library Standards in 2007, with support from a publication grant from the National Library of Medicine. Additionally, she co-edited the second edition of the Medical Library Association Guide to Managing Health Care Libraries, published in 2011. For this contribution she received the Eliot prize in 2012. Roz developed several courses, taught more than fifteen of them, and presented in over sixty invited and contributed sessions. Whenever she learned something new that could benefit other librarians, she developed an effective method of sharing that knowledge either through publications, presentations, or both.

Roz was an artist, working in pottery, watercolors, and Zentangles. She enjoyed music, theater, skiing, travel, and gardening. She volunteered at Denver's St. Francis Center for many years, providing services to individuals transitioning out of homelessness. Recent tributes, commenting on Roz's generosity and hospitality, included this story from Amy Six-Means, Clinical Research Librarian at Children's Health in Dallas, Texas. “I will always be humbled by Roz's generous spirit in sharing her home with me when I moved to Denver, not knowing me, but just because I was a librarian coming to work there. Then upon hearing me cough, after driving across the country from North Carolina, immediately took me to Urgent Care realizing I had bronchitis. Her welcoming spirit was the balm my soul needed as I started a new journey professionally and personally with that trip” [4].

Roz's life after her 2019 cancer diagnosis was as inspiring as her professional life. While undergoing treatments for the disease, she was determined to live the best life possible. She shared her journey on CaringBridge, a social network devoted to keeping families and loved ones connected during any type of health event. She spent time with family and friends and enjoyed her art and music. Roz continued her weekly COVID-era Zoom “cocktail hours” so that friends could share updates on their lives.

Most of all, Roz loved her family. Her husband, Jim Mills, her daughter, Laura Dudden, and her grandson, Julian Dudden, were her joy. Growing up in Connecticut as part of a large family, her brothers and sisters remained an essential part of her life.

All who knew Roz, worked with her, took classes from her, or read her publications are richer for what she accomplished and shared with us. Those accomplishments, and her life, will inspire others for many years to come.
